# Case report: *Echinococcus multilocularis* infection in a dog showing gastrointestinal signs in Hokkaido, Japan

**DOI:** 10.3389/fvets.2024.1373035

**Published:** 2024-06-13

**Authors:** Izumi Kida, Naoki Hayashi, Nozomu Yokoyama, Noriyuki Nagata, Kazuyoshi Sasaoka, Noboru Sasaki, Keitaro Morishita, Kensuke Nakamura, Hirokazu Kouguchi, Kinpei Yagi, Ryo Nakao, Mitsuyoshi Takiguchi, Nariaki Nonaka

**Affiliations:** ^1^Division of Risk Analysis and Management, International Institute for Zoonosis Control, Hokkaido University, Sapporo, Japan; ^2^Laboratory of Parasitology, Department of Disease Control, Faculty of Veterinary Medicine, Hokkaido University, Sapporo, Japan; ^3^Laboratory of Veterinary Internal Medicine, Department of Clinical Sciences, Graduate School of Veterinary Medicine, Hokkaido University, Sapporo, Japan; ^4^Veterinary Teaching Hospital, Graduate School of Veterinary Medicine, Hokkaido University, Sapporo, Japan; ^5^Laboratory of Molecular Medicine, Department of Disease Control, Faculty of Veterinary Medicine, Hokkaido University, Sapporo, Japan; ^6^Department of Infectious Diseases, Hokkaido Institute of Public Health, Sapporo, Hokkaido, Japan

**Keywords:** *Echinococcus multilocularis*, gastrointestinal sign, dog, zoonosis, mitochondrial genome

## Abstract

*Echinococcus multilocularis* is a cestode that causes human alveolar echinococcosis, a lethal zoonotic disease distributed in the northern hemisphere. The life cycle of this parasite is maintained in nature by voles as intermediate hosts and foxes as definitive hosts in Hokkaido, Japan. Although dogs are also susceptible to the parasite, the infection has been considered typically asymptomatic. We report the detection of *E. multilocularis* eggs in the diarrheal feces of a dog with chronic gastrointestinal signs, which disappeared after anthelmintic treatment. The mitochondrial genome sequence constructed by sequencing of the overlapping PCRs using DNA from the eggs was identical to the most predominant haplotype previously reported in red foxes in Hokkaido. This case highlights that *Echinococcus* infection should be considered as a differential diagnosis for diarrheal dogs in the disease endemic areas. Further efforts are needed to accumulate parasite genotypes in domestic dogs as well as humans to assess the risk of human infection from dogs.

## Introduction

Alveolar echinococcosis (AE) in humans, a potentially fatal zoonosis if left untreated is caused by the metacestode stage of the parasite *Echinococcus multilocularis*, which is widely distributed in the northern hemisphere. The life cycle of this parasite involves carnivores such as foxes and dogs as definitive hosts and voles as intermediate hosts. A total of 254 AE cases were reported between 2010 and 2020 in Japan, with Hokkaido having the highest burden of the disease (1). The prevalence of *E. multilocularis* infections in foxes was 30–40% in Hokkaido (2). Furthermore, studies of pet dogs in Hokkaido have estimated the infection rates to be 7.1% in a rural area (3) and 1.9% in an urban area (4). Thus, pet dogs in endemic areas may play an important role in the transmission of the parasite to humans.

The adult cestode resides in the small intestine of the definitive host, and infected hosts typically do not show clinical signs (5). However, there have been two reports of the incidental detection of *E. multilocularis* in dogs that exhibited severe gastrointestinal signs such as vomiting and diarrhea with mild hypoproteinemia (6, 7). Here, we report a case of a pet dog raised in an urban area of Hokkaido that showed intermittent gastrointestinal symptoms and was infected with *E*. *multilocularis*. When treated with an anthelmintic, the clinical signs of the dog disappeared.

## Case description

An 8-year-old female spayed Jack Russell Terrier was presented to Hokkaido University Veterinary Teaching Hospital with a 2-month history of intermittent vomiting, anorexia, and borborygmi. These clinical signs were unresponsive to symptomatic therapy provided by the referring veterinarian in Sapporo, Hokkaido. The dog had no previous clinical history, and was prescribed ivermectin monthly for heartworm prevention. The owner always kept the dog indoors or on a leash when outdoors.

**Figure 1:**
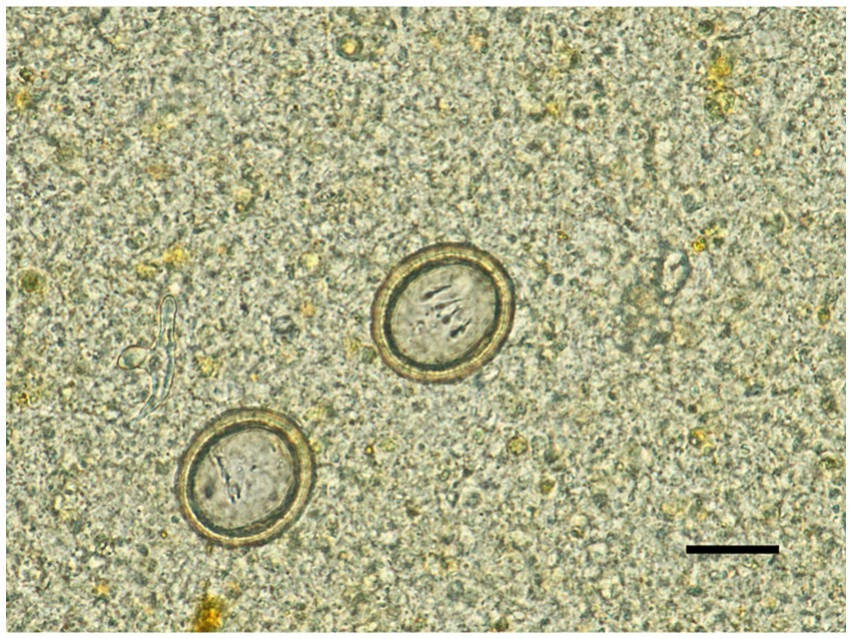
Taeniid eggs detected in a dog with gastrointestinal signs. Scale bar = 20 μm.

At presentation, the dog’s feces were watery, and blood and taeniid eggs were observed on direct fecal smear examination (Figure 1). Physical examination showed no abnormalities. A complete blood count demonstrated mild eosinophilia (1,820 cells/μL, reference range 170–1,570 cells/μL) and serum chemistry revealed no significant abnormalities. Abdominal ultrasonography revealed mild jejunal lymphadenopathy.

The dog was treated with praziquantel (6.4 mg/kg), pyrantel (18.5 mg/kg), and febantel (19.2 mg/kg) (Drontal Plus, Bayer Yakuhin, Osaka, Japan) as an oral single dose for the treatment of intestinal cestodiasis with suspected echinococcosis. The therapeutic response in the dog was carefully monitored, with chronic inflammatory enteritis (CIE) also considered as a differential diagnosis. Gastrointestinal signs resolved markedly within a few days after treatment and did not recur. A follow-up fecal examination with a flotation technique performed 5 days after treatment revealed no parasite eggs.

To identify the taeniid species of the eggs, we performed a multiplex PCR assay (8). DNA was extracted from 0.2 g of the fecal sample, according to the method previously described (9). The PCR performed using this DNA yielded a single band of ~400 bp, which was in accordance with the expected amplicon size for *E. multilocularis*.

**Figure 2:**
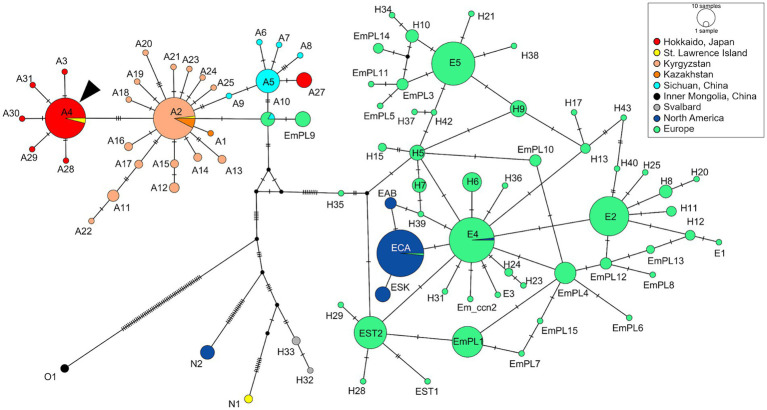
Median-joining haplotype network of *Echinococcus multilocularis* based on three mitochondrial protein-coding sequences. The analysis included haplotypes based on three mitochondrial genes (cytochrome *b*, NADH dehydrogenase subunit 2, and cytochrome *c* oxidase subunit I) previously reported in other endemic areas. The arrowhead indicates the haplotype obtained in this case (A4).

We used an amplicon-based next-generation sequencing method to genotype the parasite, targeting mitochondrial protein-coding sequences (CDSs). The whole mitochondrial genome was amplified by overlapping PCRs using four sets of primers as reported previously (10). Illumina sequencing libraries were constructed and sequenced on an Illumina MiSeq platform using the MiSeq reagent kit v3 for 600 cycles (Illumina, Hayward, CA, United States). Read mapping against a reference mitogenome sequence of *E. multiloculari*s (GenBank accession no. AB018440) (11) using CLC Genomics Workbench v20.0.4 (Qiagen, Hilden, Germany) generated a complete mitochondrial genome sequence for the parasite (accession no. LC744000). The sequence is 13,738 bp in length and consisted of 12 CDSs, 22 transfer RNA genes, and 2 ribosomal RNA genes as previously reported (10). The network analysis using PopART v1.7 (12) showed that the mitochondrial haplotype based on three complete CDSs (cytochrome *b*, NADH dehydrogenase subunit 2, cytochrome *c* oxidase subunit I) was genotype A4, which is predominantly detected in wild foxes in Hokkaido (Figure 2) (10, 13–16).

## Discussion and conclusion

We have reported a rare case of intestinal *E. multilocularis* infection in a pet dog with gastrointestinal signs. Chronic intermittent or persistent diarrhea in dogs is commonly caused by gastrointestinal or extragastrointestinal disorders. The cause of gastrointestinal disorders includes infectious, neoplastic, mechanical, toxic, or noninfectious inflammatory such as CIE. CIE is the most common cause of chronic gastrointestinal disease in dogs (79%), followed by parasitic infection (12%) (17). Given the complete resolution of these signs following anthelmintic therapy, *Echinococcus* infection was thought to be the most likely cause of the disease. In fact, experimental oral infection of *E. multilocularis* protoscoleces into beagle dogs resulted in intermittent diarrhea in two out of four dogs (18). Moreover, upon reinfection all four dogs exhibited frequent diarrhea from the early stages of the infection. These experimental data, together with previous reports of symptomatic echinococcosis in dogs (6, 7), support the assumption that dogs could develop gastrointestinal signs including diarrhea upon *E. multilocularis* infection. Thus, this parasitic disease should be considered as one of the differential diagnoses in dogs with chronic gastrointestinal signs that live in endemic areas.

Although Hokkaido is an endemic region for AE, there are no national regulations for animal deworming. The lack of regulations poses not only the risk of transmission from pet dogs to humans, but also the risk of further expansion of the parasite distribution through animal transportation (10). The case presented here, where the indoor dog became infected, underscores such potential risks of diffusion by pet animals. It would be important to apply appropriate deworming programs for high-risk dogs and to use reliable diagnostic methods.

In certain areas of Hokkaido, the periodic distribution of praziquantel-containing baits has been provided to reduce the prevalence of *E. multilocularis* in foxes. Field studies have demonstrated the efficacy of anthelmintic baiting in reducing contamination by the eggs, thereby decreasing the risk of infection (19–21). It is worth mentioning here that mass drug administration for controlling malaria has been associated to the emergence of drug-resistant parasites (22–25). Although the drug resistance in *E. multilocularis* has never been reported, a recent study introduced the clinical resistance to praziquantel in another cestode, *Dipylidium caninum* (26). Therefore, it is important to monitor the drug resistance in clinical settings and explore alternative treatment options for canine echinococcosis.

Genotyping parasites is essential for a better understanding the epidemiological status of infectious diseases. For instance, a previous study in Kyrgyzstan found that haplotype A2 was the most common genotype in both humans and dogs, which is pivotal information to analyze the transmission dynamics and pathogenicity of the parasites (16). Previous research has revealed that an Asian haplotype A4 is the most predominant haplotype among red foxes in Hokkaido (10). The genotype detected in the current study was also assigned to the same haplotype, which may indicate that dogs are likely to have the same susceptibility to the genotype as foxes. Given the potential phenotypic differences among genotypes (27, 28), further studies genotyping parasites affecting both pet dogs and humans would be valuable to assess the risk of human infection from dogs.

In the current case, the animal was always kept indoors or on a leash when outdoors, and never observed any hunting behavior. These are contrary to the risk factors for canine echinococcosis, including roaming outdoors unattended and hunting/killing small animals (29, 30). Nevertheless, the dog likely became infected as a result of ingesting infected vole(s) during outdoor activities, such as preying or scavenging. This is supported by the facts that the detected parasite was the most dominant haplotype in Hokkaido, and the dog had no travel history outside Hokkaido. Taken together, this case emphasizes the importance for pet owners and animal care professionals to understand the risk of transmission of *E. multilocularis* even from indoor dogs in the endemic regions. It also highlights the need to employ proper biosafety protocols when handling suspect animals showing gastrointestinal signs.

## Data availability statement

The datasets presented in this study can be found in online repositories. The names of the repository/repositories and accession number(s) can be found in the article/supplementary material.

## Ethics statement

The requirement of ethical approval was waived by Ethics Screening Committee of Hokkaido University Veterinary Teaching Hospital for the studies involving animals because this is a case report of examinations performed for the purpose of patient treatment, and no action contrary to treatment was performed. The studies were conducted in accordance with the local legislation and institutional requirements. Written informed consent was obtained from the owners of the animals for the publication of this case report.

## Author contributions

IK: Funding acquisition, Investigation, Writing – original draft. NH: Formal analysis, Investigation, Methodology, Writing – original draft. NY: Conceptualization, Supervision, Writing – review & editing. NoN: Resources, Writing – review & editing. KS: Resources, Writing – review & editing. NS: Resources, Writing – review & editing. KM: Resources, Writing – review & editing. KN: Resources, Writing – review & editing. HK: Resources, Writing – review & editing. KY: Funding acquisition, Resources, Writing – review & editing. RN: Conceptualization, Supervision, Writing – review & editing. MT: Resources, Supervision, Writing – review & editing. NaN: Conceptualization, Funding acquisition, Supervision, Writing – review & editing.
